# Computational approaches for drug repurposing in oncology: untapped opportunity for high value innovation

**DOI:** 10.3389/fonc.2023.1198284

**Published:** 2023-05-18

**Authors:** Shraddha M. Dalwadi, Andrew Hunt, Mark D. Bonnen, Yohannes T. Ghebre

**Affiliations:** Department of Radiation Oncology, The University of Texas Health Science Center at San Antonio, San Antonio, TX, United States

**Keywords:** drug repurposing, pharmacology, oncology, informatics, clinical trials

## Abstract

Historically, the effort by academia and industry to develop new chemical entities into lifesaving drugs has limited success in meeting the demands of today’s healthcare. Repurposing drugs that are originally approved by the United States Food and Drug Administration or by regulatory authorities around the globe is an attractive strategy to rapidly develop much-needed therapeutics for oncologic indications that extend from treating cancer to managing treatment-related complications. This review discusses computational approaches to harness existing drugs for new therapeutic use in oncology.

## 1 Introduction

Drug development takes time and often has limited success in meeting the demands of today’s healthcare. This is in major part due to the length of time it takes to bring new drugs to the market, the staggering cost of *de novo* drug development, and the high rate of attrition during development (1). Current estimates of drug development indicate that it takes over 12 years of time and over $100 million US dollars to develop new chemical entities (NCEs) into actual drugs (2).

**Figure 1:**
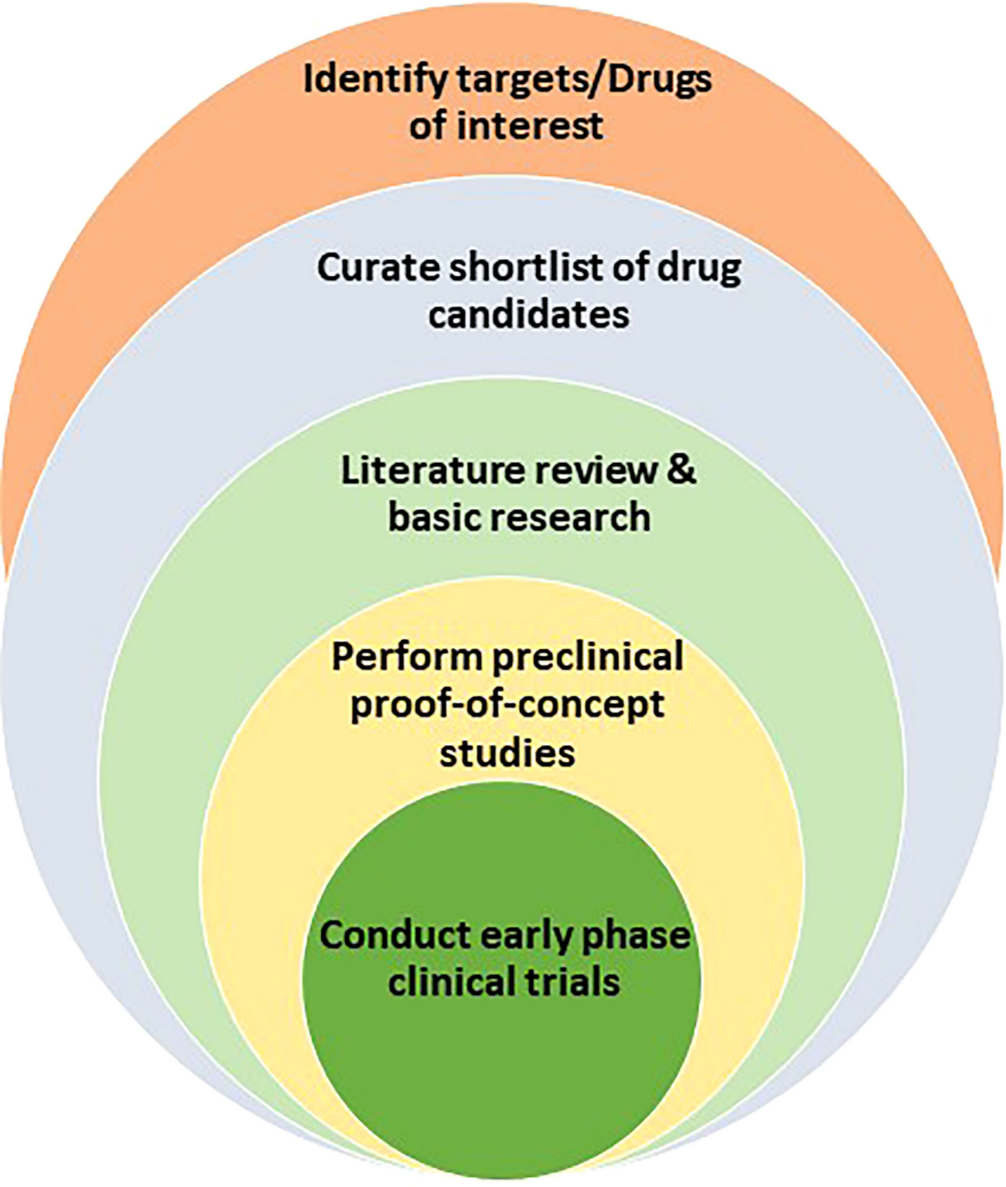
Drug repurposing path that encompasses the identification of drug targets or drug candidates, the curation of promising candidates, the leveraging of literature review and basic research to test and validate hypothesis, the performance of proof-of-principle *in vivo* preclinical studies, and the launch of clinical trials for new indications.

Even after the investment of such resources, less than 2% of the NCEs are developed into drugs (98% attrition). The major contributors for the failure of drugs-in-development are lack of safety and efficacy (3). After pre-clinical study to establish feasibility, NCEs must pass the rigor of phase I and II trials to establish favorable toxicological and pharmacological profiles in the clinical setting. The few drug candidates that survive the scrutiny of phase I and II clinical trials proceed to phase III trials to validate their clinical efficacy in a large number of patients who present various stages of a given disease and comorbidities. Mitigating the uncertainties that surround *de novo* drug discovery and development, and streamlining the clinical trial process are necessities in oncology, as cancer remains to be a major public health problem worldwide. One possible solution to the soaring public health crisis is the repurposing and repositioning of existing drugs for new indications in oncology (**Figure 1**). This review discusses computational approaches to harness existing drugs for new therapeutic use in oncology.

## 2 Understanding drug repurposing

The idea of drug repurposing (new use of existing drugs), and drug repositioning (re-investigation of drug candidates that failed approval for one indication) goes beyond simply considering conventional drugs for new uses (4). The notion of repurposing Food and Drug Administration (FDA)-approved drugs, for example, is embedded in the concept that when a given molecular target interacts with a drug, it often regulates numerous anticipated and unexpected biological responses. The polypharmacology of on- and off- target effects of drugs can be capitalized upon even in cases where the biological process is not completely understood. In this regard, multiple computational approaches are emerging to assist in the identification and repurposing of drugs. These bioinformatics tools explore the opportunities to repurpose or reposition drugs from at least three perspectives: new target-old indication, new target-new indication, and old target-new indication.

After a comprehensive list of candidate drugs is curated from existing chemical libraries, the next step is to gather supporting evidence and perform *in silico* screening for pre-defined parameters, including pharmacologic and toxicologic properties. A handful of drugs are then selected for proof-of-concept studies using molecular, cell biological and preclinical studies. Once favorable preclinical data is generated, early phase clinical study can be designed and conducted in healthy volunteers and/or defined patient population. Here, most drugs that are originally approved by the FDA may qualify for Investigational New Drug (IND) exemption which would accelerate clinical testing (5). Drugs that involve reformulation (i.e. change in the route of administration), dose adjustment, or compound mixing may ultimately require FDA involvement prior to human study, though the regulatory process is generally much easier than the path to *de novo* drug development. Once the issue of IND is cleared, a series of clinical studies would determine the safety and therapeutic efficacy of the candidate drugs. Finally, if the drug is found to be safe and efficacious in treating a new oncologic disease or mitigating a complication of standard cancer therapy, the FDA may approve the drug for the new indication and incorporate into oncologic practice.

## 3 Emerging computational approaches

**Figure 2:**
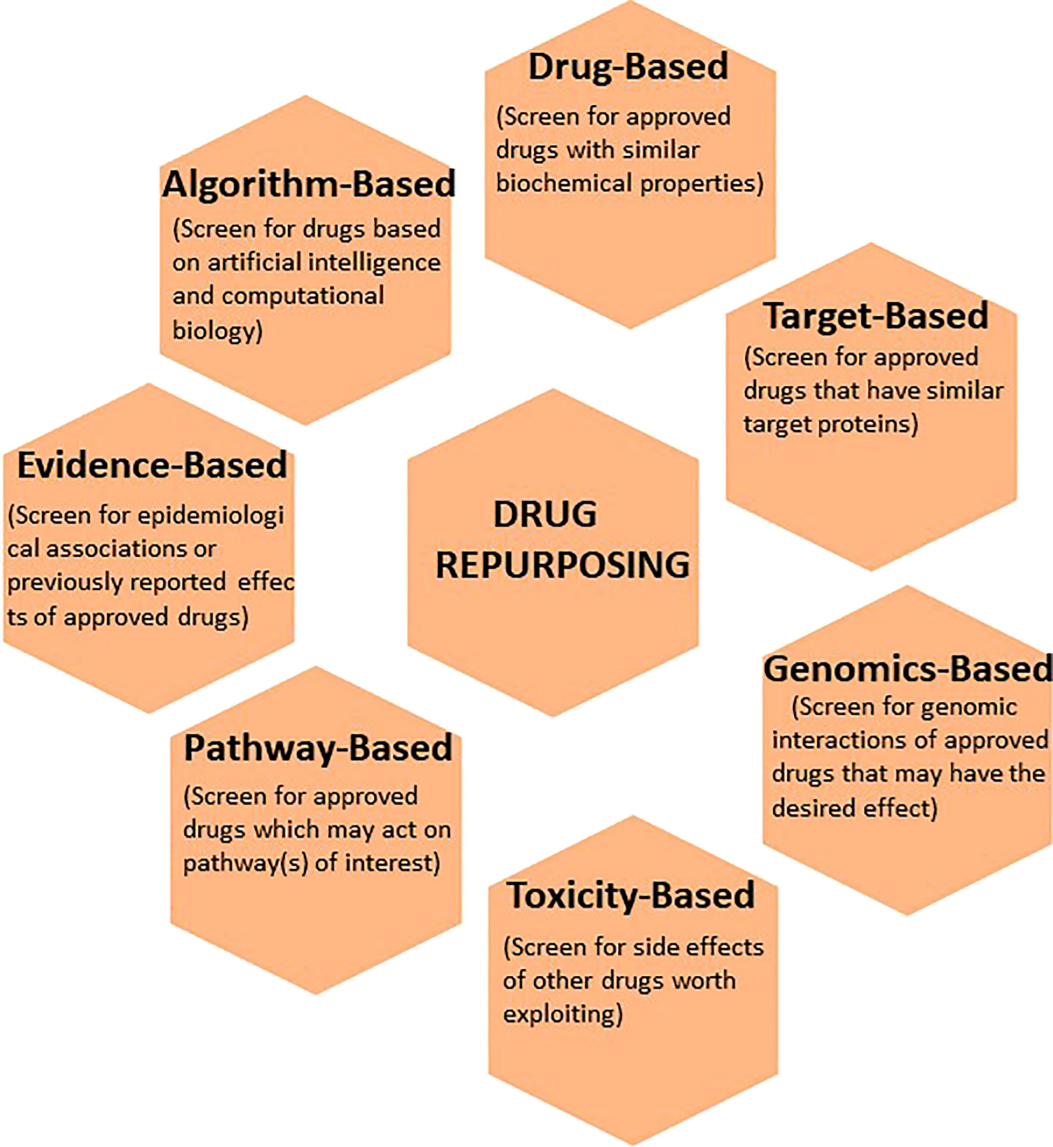
The application of various computational techniques to enhance the discovery and development of existing drugs for new indications. Commonly used informatics tools that utilize drug-based, target-based, genomics-based, toxicity-based, pathway-based, evidence-based, and algorithm-based approaches to drug repurposing are illustrated.

Advancement in the organization and communication of health information through technology has laid the foundation for multiple widely-available computational approaches to develop a pipeline for curating a list of drug candidates, testing hypothesis-driven concepts, as well as generating new hypotheses to identify new uses of existing drugs. Various databases and software algorithms now increase the throughput and diminish the time required to determine repurposing potential. Some of these bioinformatics tools are open access and freely available to the public. Moreover, the rapidly expanding “omics” databases allow for interrogation of biological processes which may otherwise not have been apparent. Below is a description of various computational approaches to identify and develop existing drugs for new indications (**Figure 2**).

### 3.1 Drug profile-based

One obvious strategy is to investigate existing drugs for the biochemical properties of interest. In this regard, the National Library of Medicine’s Drug Information Portal is an online dictionary that could be utilized when initially searching for drugs-of-interest (6). In addition, online encyclopedias, such as PubChem, allow the search of information including chemical and physical properties, biological activities, safety and toxicity profiles, patents, and relevant literature (7). In these databases, compounds can be browsed by molecular structures or chemical formulas. DrugBank is a similar database that incorporates pharmacologic information of several existing drugs (8). Moreover, the FDA Online Drug Label Repository can be utilized for relevant disclosed information and official FDA position on active pharmaceuticals (9). As the outcome of this approach depends on the input data, private companies have also developed their own drug databases, promising superior data and search technology.

### 3.2 Toxicity-based

Known or documented side effects of drugs can be exploited for drug repositioning or repurposing in oncology. Pharmacovigilance dictionaries that can be queried include the Adverse Drug Reaction Database, FDA Adverse Event Reporting System (FDAERS), and Side Effect Resource (SIDER). Toxicology in the 21^st^ Century (Tox21) Consortium is a concerted effort by the Environmental Protection Agency (EPA), National Institute of Health (NIH), and the FDA to assist researchers in improved toxicity assessment. Currently, the National Center for Advancing Translational Sciences is employing a high throughput screening platform known as Tox21 10K Library to understand the potential of chemicals in disrupting biological processes (10). Toxicology Forecaster (ToxCast) is yet another initiative by the EPA that was established to identify and trace biological pathways involved in side effects so that they can be harnessed for therapeutic purposes (11). With large volumes of data, researchers now can identify, either retrospectively or prospectively, potential toxicities based on the molecular structure of the target of interest and help determine a preclinical toxicity threshold prior to any trials with patients (12).

### 3.3 Genomics-based

Over the past decade, it has become evident that understanding the intricacies of the human genome is a critical component of determining cancer risk, disease severity and clinical management (13). With the widespread implementation of genome sequencing in healthcare, multiple databases are available to help determine such relationships. Among these are: Sequence Read Archive (SRA), Gene Expression Omnibus (GEO), Single Nucleotide Polymorphism Database (dbSNP), and Online Mendelian Inheritance in Man (OMIM). In this regard, software algorithms that can be used to analyze genomic data of interest include Genome Wide Association Studies (GWAS) and Connectivity Map (CMAP). Finally, CRISPR-based technology has allowed researchers to test specific genomic targets at a scale, thus increasing the pace of drug development (14).

### 3.4 Target-based

Disease-driving molecular targets can be directly examined to evaluate if the targets are engaged by drugs that have not been previously known to interact with (15). There are several resources to aid with target-based interactions. For example, proteins that are encoded by any human gene can be reviewed in the Human Protein Reference Database. Structural and functional protein-protein interactions are available in the STRING database, along with their basis (computational prediction or a primary source). The Therapeutic Targets Database (TTD) has information regarding therapeutic targets with references to disease and associated drugs. This approach was used to find existing drugs that were candidates for treatment of Candida albicans in the setting of multidrug resistance (16).

### 3.5 Pathway-based

While individual genes/proteins can be pharmacologically targeted, drugs that broadly engage biological processes can essentially accomplish the intended effect and therefore should be closely investigated for their pharmacologic potential (17). In this regard, the Reactome Pathway Disease is a visual bioinformatics tool that can be utilized in analyzing signaling pathways under physiological and pathological conditions. Other resources such as Pathway Commons, Pathway Interaction Database (PID), and the Kyoto Encyclopedia of Genes and Genomes (KEGG) are also available for pathway-based computational studies. As an example of this approach, 54 new drug candidates for the treatment of Alzheimer’s were recently discovered (18).

### 3.6 Evidence-based

While investigating new uses of existing drugs on the basis of biological pathways, drug profile, and retrospective clinical data is a prudent approach, some relationships are not completely understood at the molecular level (19). For example, the association of metformin and statins with reduced risk of malignancy is well-documented in epidemiologic studies. In fact, many prospective clinical trials are evaluating these drugs in cancer patients to assess therapeutic efficacy. Similarly, other FDA-approved drugs with similar anecdotes should be profiled and investigated in preclinical studies, as well as clinical databases (such as The Surveillance, Epidemiology, and End Results (SEER)-Medicare linked database) to determine the potential for repurposing.

### 3.7 Algorithm-based

Finally, the approaches above have been amplified by artificial intelligence and computational biology. Specifically, machine learning can be used to predict drug efficacy, toxicity, and dose. In this approach, large datasets are fed into an algorithm (e.g. random forests) to determine initial candidates. These candidates can be identified based on existing chemical scaffolds or can be empirically designed as potential novel drugs that engage defined molecular targets. Subsequently, a neural network can be used to construct knowledge graph that is based on a drug’s biological interactions. Recently, computational approaches have been used to model the molecular structure of the SARS-CoV-2 viral proteins, and speed up the development of COVID 19 drugs and vaccine candidates (20, 21).

### 3.8 Examples of successfully repurposed and repositioned drugs

Over the past 25 years, there are several examples of successfully repurposed and repositioned drugs for a number of new indications (22). Among these are antidepressant drugs that found new use for non-neurological indications. For example, bupropion (Wellbutrin) was developed by GlaxoSmithKline (GSK) for the treatment of depression. The same company repurposed the drug, renamed as Zyban, for the cessation of smoking. Intriguingly, the repurposing did not involve any change in formulation or dose, and Zyban grosses over $125 million dollars per year in revenue. Another example of a successfully repurposed antidepressant drug is the selective serotonin reuptake inhibitor (SSRI) Dapoxetine. The drug was originally developed by Eli Lilly as an analgesic and for the treatment of depression. However, it was found to be ineffective for these indications. Surprisingly, Dapoxetine (branded as Priligy) was successfully developed for the treatment of premature ejaculation and is now a $200 million per year drug for this indication. Other examples of successfully repurposed antidepressant drugs include Duloxetine (Cymbalta), a non-selective serotonin reuptake inhibitor, that was repurposed for the treatment of stress urinary incontinence (SUI) that is currently marketed (over $800 million annual revenue) under the brand name Duloxetine SUI; and Fluoxetine (Prozac), an SSRI that was repurposed under the brand name Sarafem for the treatment of premenstrual dysphoria (over $60 million annual revenue). There are also several examples of successfully repurposed non-psychoactive drugs. Among these are Propecia, the widely used hair loss treatment that was originally developed for the treatment of enlarged prostate (Proscar); and Minoxidil, an antihypertensive drug that was repurposed as Rogaine for the treatment of hair loss. Interestingly, the revenue of Propecia and Rogaine by far exceed that of the originally developed respective drugs, Proscar and Minoxidil respectively. Examples of drugs that are successfully repurposed for oncologic indications include thalidomide (Contergan), a drug originally developed for the treatment of morning sickness and is now marketed for the treatment of multiple myeloma (marketed under the brand name Thalomid). Recently, we have taken the initiative to reformulate and repurpose the antacid drug Esomeprazole (Nexium) for the treatment of radiation dermatitis (23, 24). Currently, this study is in phase II clinical trial for the new indication in patients with head and neck cancer (NCT04865731).

In addition to the aforementioned success stories in drug repurposing, drug repositioning has also had its success. The most striking story of drug repositioning has been that of Sildenafil; a drug that failed the original indication for the treatment of angina but became a blockbuster drug (marketed as Viagra) for the new indication of erectile dysfunction (ED). Ironically, this drug is now a $1.88 billion dollar per year enterprise as a repositioned drug for ED.

Taken together, there are scopious examples of successfully repurposed and repositioned drugs that should be leveraged to stimulate deep interest among the academic institutions and biopharmaceutical companies that strive to add new armament of drugs to their anticancer arsenal. The substantially reduced timeline and cost in conjunction with the higher probability of success and attractive revenue stream should make drug repurposing and repositioning efforts worthwhile.

## 4 Conclusion

Repurposing drugs that are originally approved by the United States FDA or the FDA-equivalent regulatory authorities around the world is an attractive strategy to rapidly develop much needed drugs for oncologic indications that extend from treating cancer to managing complications that are induced by the current anticancer drugs or radiation therapy. There are significant incentives that are embedded in the concept of drug repurposing to include the rapid path to development (3-5 years on average), the cost effectiveness (under $10 million US dollars), and the significant success rate (over 12-fold greater than *de novo* drug discovery and development). Paradoxically, drug repurposing and repositioning remain underutilized in oncology. It is possible that the staggering healthcare expenses including the cost of emerging anticancer drugs will urge us to aggressively utilize computational approaches and high throughput drug screening platforms to recycle existing drugs for new oncologic indications. In this regard, we have assembled available resources for investigators who are interested in pursuing drug repurposing to uncover new uses of existing drugs.

## Author contributions

SD, MB, and YG contributed to conception and design of the manuscript. SD wrote the first draft of the manuscript. SD, AH, MB, and YG contributed to manuscript revision. All authors contributed to the article and approved the submitted version.
